# Anaesthetic management of closed mitral valvotomy for severe mitral stenosis with traumatic kyphoscoliosis

**DOI:** 10.4103/0019-5049.60502

**Published:** 2010

**Authors:** AM Jagadeesh, N Manjunath, Venugopal Ram Rao, Sunitha A Sathyakumari

**Affiliations:** Department of Anesthesiology, Sri Jayadeva Institute of Cardiology, Bangalore, India

**Keywords:** Closed mitral valvotomy, kyphoscoliosis, mitral stenosis, osteoarthritis, percutaneous transluminal mitral commisurotomy

## Abstract

A 42-year-old male patient with rheumatic mitral stenosis was posted for percutaneous transluminal mitral commissurotomy. He had associated traumatic kyphoscoliosis and osteoarthritis of hip and knee joints, causing severe permanent flexion of these joints. This position caused technical difficulty in approach to the femoral vessels. So he was rescheduled for closed mitral valvotomy. This also posed similar problems, but was successfully managed.

## INTRODUCTION

Mitral stenosis is almost always due to fusion of the mitral valve leaflets at the commissure during the healing process of acute rheumatic fever.[1] Patient is symptomatic when the size of mitral valve orifice area is less than 1 sq cm.[2] The most definite assessment of the degree of mitral-stenosis is done by measuring the diastolic gradient. Any gradient more than 10 mm of Hg suggests severe stenosis.

Associated kyphoscoliosis, which is a defomity of the costo-vertebral skeletal structure, is characterized by an anterior flexion (kyphosis) and lateral curvature (scoliosis) of the vertebral column. A curve more than 40° is considered severe and most likely associated with physiological derangement in cardiac and pulmonary function. Restrictive lung disorder and pulmonary hypertension[3] progressing to corpulmonale are the principle causes of mortality in these patients[4].

We report a case of rheumatic mitral stenosis with concomitant severe kyphoscoliosis undergoing closed mitral valvotomy.

On examination he had pulse rate of *84/minute,* regular, blood pressure 110/70 mm of Hg. Cardiac auscultation revealed S 1 loud, mid diastolic murmur, opening snap and loud P2. There was significant Kyphoscoliosis at T8- L4 with prominence of spines. Osteoarthritis of Hip joint, Knee joints and cervical vertebrae present, which was restricting his extension movement and preventing him from walking straight or lying down flat.

A 42-year-old male patient presented with complaints of palpitation, easy fatigue, and breathlessness, loss of appetite since one year. Significant past history was that of a motor vehicle accident injuring his back bone at the age of 15 years, for which he was treated only with medicine as he refused surgery on financial grounds. However, he noticed progressive forward bending of the back bone in later years.

Blood investigation was within normal limits. ECG showed sinus rhythm with ‘p’ mitrale in V-1. Chest x-ray revealed deviation of trachea slightly toward right side and crowding of ribs with mitralisation of the left border of the heart. 2D echocardiogram showed severe mitral stenosis, Mitral valve orifice area = 0.8 cm^2^, Sub mitral fusion and mitral valve gradient = 35/23 mm of Hg, Pulmonary artery systolic pressure = 58 mm of Hg. Pulmonary function test revealed restrictive lung disorder.

Patient was taken up for percuteneous translurninal mitral comrnissurotomy.[5] However, the procedure was abandoned due to the following reasons:

1. Patient was unable to lie down in supine position due to severe kyphoscoliosis.
2. Extreme flexion of hip and knee joints caused total inaccessibility to approach the femoral vessels. Hence closed mitral valvotomy 2·7 was undertaken for this patient.

On preanaesthetic evaluation, patient was graded as ASA Grade IV, Mallampati Grade 1 and neck extension was restricted. He was premedicated with oral Diazepam 10 mg, Benadryl 25 mg. Patient was shifted to operation theatre with 18G venous canula. Intra arterial blood pressure, 12 lead ECG, SP0_2_, ETC0_2_, and nasopharyngeal temperature was monitored. Difficult airway anaesthetic drill including laryngeal mask airway and fibreoptic laryngoscope were kept ready.

**Figure 1:**
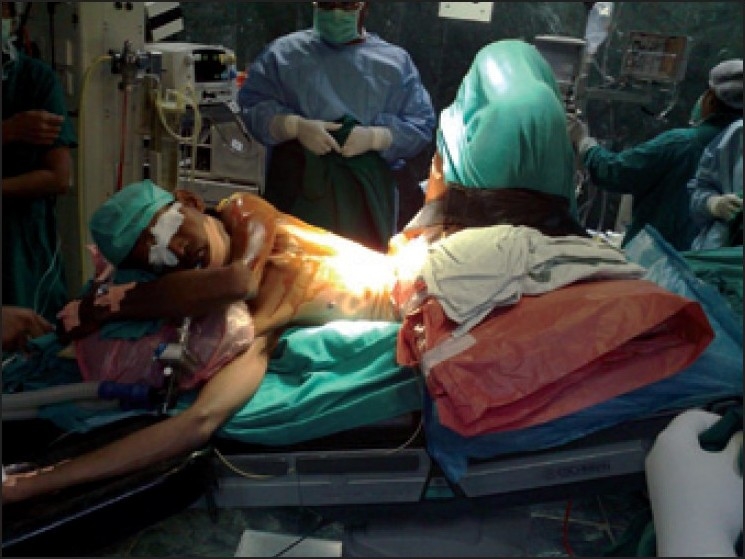
Photo of the patient showing steep Trendelenburg position after intubation

Patient was induced with Midazolam- 0.1 mglkg, Glycopyrrolate 0.2 mg, Fentanyl-3 mcg/kg, and rocuronium- 0.5 mglkg and intubated with 9.5 mm endotracheal tube. He was positioned in left lateral decubitus position with three pillows to support his neck. Both the legs were extremely flexed at hip and knee joints. This position was difficult for surgeons to approach the 5th intercostal space. Therefore, steep Trendelenberg position was given for better exposure [Figure 1]. This resulted in increased preload which necessitated the introduction of positive end expiratory pressure to prevent pulmonary oedema.

The surgical approach was through left anterolateral thoracotomy at 5^th^ inter costal space. Mitral valve opened up to 2.5 cm^2^ through left ventriculotomy using Tubb's dilator,[7] and no peroperative Mitral regurgi tation detected. Intraoperative anaesthetic maintenance was with inhalation Isoflurane, intravenous Fentanyl, midazolam, Rocuronium. Intercostal nerve block was given for postoperative pain management using 10 ml Bupivacaine 0.25%. Intraoperative haemodynamic status was maintained with fluids and dopamine, patient was shifted to recovery room and ventilated for four hours.

Patient was extubated when all the clinical parameters and arterial blood gases were normal and nasal O_2_ connected. Patient had CO_2_ retention on the same night, which was managed with BiPAP mask, nebulisation and good chest physiotherapy. Postoperative period was uneventful and he was discharged on 10^th^ day.

**Table:** 

	Preoperative	Postoperative
MVOA	0.8 cm^2^	2.3 cm^2^
MV gradient	35/23 mmHg	7/4 mm Hg
PASP	58 mm Hg	27 mm Hg

## DISCUSSION

In kyphoscoliosis, a curve more than 40° is considered severe, which results in derangement of cardiac and pulmonary function. Restrictive lung disease and pulmonary hypertension progressing to corpulmonale are the principal causes of mortality. As the curve worsens, more lung tissues are compressed, resulting in decreased vital capacity^6^ and dyspnoea with mild exertion. The work of breathing is increased by abnormal mechanical properties of the thorax, by increased airway resistance resulting from a small lung volume, the alveolar- to- arteriolar difference for O_2_ is increased.

Our patient, with symptomatic mitral stenosis and concomitant severe kyphoscoliosis, was referred for PTMC. It was technically challenging because of multiple anatomical aberrations, which distort the spatial relationship of the intracardiac and neighbouring structures,[3] making trans septal puncture potentially hazardous in terms of an increased risk of inadvertent cardiac perforation, Tamponade, difficulty in resuscitating the patient and even death. Thoracic spinal deformity has hence been traditionally considered a contraindication to the PTMC procedure. In this case, extreme flexion of the hip and knee joints due to osteoarthritis posed positioning problem and inaccessibility to femoral vessels. Mitral stenosis with severe kyphoscoliosis may pose positional embarrassment to both surgeons and anaesthetists.

They are induced and maintained in head-up position. Any head-down position may lead to increase in preload, pulmonary congestion and pulmonary oedema. This patient had to be positioned in steep trendelenburg position because of kyphoscoliosis. Steep Trendelenburg position after anaesthesia would cause increased venous return and pulmonary congestion. A high positive end expiratory pressure would cause haemodynamic embarrassment and low cardiac output. Hence optimal PEEP of 6cm of H_2_O was introduced. Serial blood gases were measured throughout the procedure. Closed mitral valvotomy was successful and the patient's endotracheal tube was removed after four hours.

In conclusion, there were multiple problems in this patient, which were handled with prior planning to bring out a successful outcome.

## References

[1] Sivasubramanian M (1997). Mitral stenosis: Surgical option. Heart Dis.

[2] Frazer K, Tumar MA, Sugden BA (1976). Closed mitral valvotomy. Br Med J.

[3] Lau KW, Ding ZP, Lee CY, Koh TH, Gao W, Johan A (1996). Technically demanding Inoue-balloon mitral commissurotomy: Broadened indications for the procedure. Singapore Med J.

[4] Ben Farhat M, Ayari M, Maatouk F, Betbout F, Gamra H, Jarra M (1998). Percutaneous balloon versus surgical closed and open mitral commissurotomy: Seven-year follow-up results of a randomized trial. Circulation.

[5] Patel JJ, Shama D, Mitha AS, Blyth D, Hassen F, Le Roux BT (1991). Balloon valvuloplasty versus closed commissurotomy for pliable mitral stenosis: A prospective hemodynamic study. J Am Call Cardiol.

[6] Stoelting RK, Dierdof SF (1988). Anesthesia and coexisting disease.

[7] Chikwe J, Walther A, Pepper J (2004). The surgical management of mitral valve diseases. Br J Cardiol.

